# Downregulation of both gene expression and activity of Hsp27 improved maturation of mouse oocyte in vitro

**DOI:** 10.1186/1477-7827-8-47

**Published:** 2010-05-14

**Authors:** Jin-Juan Liu, Xiang Ma, Ling-Bo Cai, Yu-Gui Cui, Jia-Yin Liu

**Affiliations:** 1 Department of life science and technology, China Pharmaceutical University, Nanjing 210038, China; 2 Center of Clinical Reproductive Medicine, First Affiliated Hospital, Nanjing Medical University, Nanjing, 210029, China

## Abstract

**Background:**

Heat shock protein 27 (Hsp27), a member of the small heat shock protein family, is an apoptosis regulator. Our previous proteomic study showed that Hsp27 mainly expressed in human oocyte, and that Hsp27 expression was downregulated in the ovaries derived from women with the polycystic ovary syndrome (PCOS), a well known endocrinal disorder with abnormal apoptotic activity and folliculogenesis. However, the exact effects of Hsp27 downregulation on oocyte development have not yet been clarified.

**Methods:**

The expression of Hsp27 gene was downregulated in the mouse oocytes cultured in vitro using siRNA adenovirus infection, while the activity of Hsp27 was decreased by microinjection of polyclonal Hsp27 antibody into the cytoplasm of germinal vesicle (GV) oocytes. Oocyte maturation rate was evaluated by morphological observation. Early stage of apoptosis was determined using Annexin-V staining analysis and some critical apoptotic factors and cytokines were also monitored at both mRNA level by real time RT-PCR and protein expression level by immunofluorescence and western blot.

**Results:**

Hsp27 expressed at high level in maturing oocytes. Infection with AdshHsp27, and microinjection of Hsp27 antibody into GV oocytes, resulted in the improved oocyte development and maturation. Germinal vesicle breakdown (GVBD) rates were significantly increased in two AdshHsp27-treated groups (88.7%, 86.0%) and Hsp27 antibody-injected group (77.0%) when compared with control (76.2% in AdGFP, 64.4% in IgG-injected), respectively. In addition, the rates of metaphase II (MII) development in two AdshHsp27-treated groups (73.8%, 76.4%) and Hsp27 antibody-injected group (67.3%) were higher than that in the controls (59.6% in AdGFP, 55.1% in IgG-injected). We also found that the rates of early stage of apoptosis in Hsp27 downregulated groups (46.5% and 45.6%) were higher than that in control group (34.1%) after 8 h of IVM. Similarly, downregulation of Hsp27 caused a significantly enhanced the expression of apoptotic factors (caspase 8, caspase 3) and cytokines (bmp 15 and gdf 9).

**Conclusions:**

Downregulation of Hsp27 improved the maturation of mouse oocytes, while increased early stage of apoptosis in oocytes by inducing the activation of extrinsic, caspase 8-mediated pathway.

## Background

Polycystic ovarian syndrome (PCOS) is known as one of the most common endocrine disorders affecting approximately 5%-10% of women of reproductive age, and is characterized by chronic anovulation, hyperandrogenism and polycystic change in ovaries [1-4]. Accumulation of small antral follicles arrested in their development, with some atretic features, has been shown in ovaries subjected to PCOS [5-9]. Those atretic follicles were closely related to inside oocyte competence [10-12]. In addition, oocyte developmental competence was susceptible to derangement in PCOS, indicating that abnormal oocyte competence in PCOS was inextricably linked to abnormal follicular development [13-17].

In the ovary, apoptosis has been implicated in the granulose cells of atretic antral follicles and in regressing corpora lutea [18-22]. Derangement of apoptotic activity was observed in PCOS ovary tissue with the altered expression of apoptotic-related regulators, including heat shock proteins (Hsp 90, Hsp 10), nuclear receptor subfamily, dickkopf homologue 3, and so on [23-26]. Hsp27, belonging to the small heat shock protein family, is a molecular chaperone protein involved in cellular protection in response to a variety of stresses such as heat shock, toxicants, injury, and oxidative stress [27-30]. Emerging evidences show that Hsp27 has strong anti-apoptotic properties by interacting directly with the caspase activation components in apoptotic pathways, consequently exerting protective effects in apoptosis-related injuries [31-34]. Interestingly, our previous proteomic study showed that Hsp27, a strong anti-apoptotic regulator, mainly localized in human oocyte, and was downregulated in the ovaries derived from women with PCOS [35]. However, the alteration of apoptotic activity, as well as effect of Hsp27, in PCOS ovaries needs to be further clarified.

Our hypothesis was that Hsp27 and its related pathways could have some effects on oocyte development, maturation, apoptosis and cell cycle *in vivo *and *in vitro*, and even participate in the follicle development and PCOS pathophysiology. In this pilot study, we firstly investigated the effect of Hsp27 downregulation on the meiotic progression and apoptosis in mouse oocyte model cultured in vitro.

## Methods

### Animals

The ICR mice were fed *ad libitum *with a standard diet and maintained in a temperature and light-controlled room (20-22°C, 12/12 h light/dark), in accordance with the Animal Research Committee Guidelines of Nanjing Medical University.

### Collection and culture of mouse oocytes

Germinal vesicle (GV) oocytes were collected from 6-week-old female ICR mice. 46-48 h previously, mice were received an intraperitoneal injection of 10 IU of pregnant mare serum gonadotropin (PMSG, Folligon, Intervet, Castle Hill, Australia). Mice were sacrificed, and ovaries were placed in M2 medium (Sigma, St. Louis, MO). Cumulus oocyte complexes were recovered from ovaries by repeatedly puncturing the surface with fine steel needles, and cumulus cells were removed by hyaluronidase treatment (Sigma, 300 U/ml in PBS) under a dissecting microscope [36]. For preparation of zona pellucida-free oocytes, the oocytes were then exposed to acidic Tyrode's solution (pH 2.5-3.0) with aspiration of the oocyte in and out of a glass micropipette to remove the zona pellucida [37]. Usually the zona pellucida was only partially dissolved and it could be removed by the pipette within 30s. Ding *et al *reported that zona-free oocytes didn't affect normal fertilization and blastocyst development [38]. After being immediately transferred to M2 and washed three times, 20-25 zona-free oocytes per group were put into 32 μl droplets of maturation medium of M16 (Sigma) containing 10% fetal bovine serum (FBS; Invitrogen, Grand island, NY) in a 5% humidified atmosphere at 37°C under a layer of mineral oil. These prepared zona-free oocytes were then used in either control or treatment groups for further investigation.

### Recombinant adenovirus generation and infection

The complementary DNA sequence of Hsp27 was obtained from GenBank (accession no. NM_013560). The potential target sequences for RNA interference (RNAi) were scanned with the siRNA Target Finder and Design Tool available from the Ambion Website [39]. The selected target sequences (shHsp27(1) and shHsp27(2)), 5'-GATCCCCGCTGGGAAGTCTGAACAGTTTCAAGAGAACTGTTCAGACTTCCCAGCTTTTTGGAAA-3'(sense) and 5'-GATCCCCCATGGCTACATCTCTCGGT TTCAAGAGAACCGAGAGATGTAGCCATGTTTTTGGAAA-3'(sense), corresponded to region 541-559 bp and 736-754 bp after the Hsp27 start codon, respectively. The negative control (randomized sequence) was: 5'-GATCCCCCATGG CTAATCCGTTCTGCTTCAAGAGAGCAGAACGGATTAGCCATGTTTTTGGAAA-3'[40,41]. These sequences were subcloned into pShuttle-H1 according to the method used by Shen *et al *[42]. The pShuttle-H1-siRNA/Hsp27 was then recombined with backbone pAdEasy-1 in BJ5183 bacteria. Adenovirus generation, amplification and titer examinations were performed according to the simplified system described by He *et al*. [43]. Viral titer was determined by plaque assay in 293 cells.

Zona-free oocytes were incubated with adenovirus at the same multiplicity of infection (MOI) in medium at 37°C for specific durations as indicated in the following experiments.

### Microinjection of Hsp27 antibody into GV oocytes

Hsp27 antibody (stock solution, 200 ug/ml, goat polyclonal, Santa Cruz Biotechnology, Santa Cruz, CA) was microinjected into the cytoplasm of GV zona-intact denuded oocytes as described by Dai *et al *[44]. Goat normal IgG-injected (Santa Cruz, CA) oocyte and untreated oocytes were used as controls to assess injection itself damage. To determine maturation rate *in vitro*, the microinjected GV oocytes were cultured in M16 medium (Sigma) in a 5% CO_2 _incubator at 37°C for 14 h. Oocytes without GVs were scored as germinal vesicle breakdown (GVBD) stage, while those with a polar body scored as metaphase II (MII). The microinjection experiments were done 10-12 replicates, using a total of > 300 oocytes. A Nikon Diaphot ECLIPSE TE 300 inverting microscope (Nikon UK Ltd, Kingston upon Thames, Surrey, UK) equipped with Narishige MM0-202N hydraulic three-dimensional micromanipulators (Narishige Inc., Sea Cliff, NY) was used. Microinjection was completed in 40 min, with volume of about 5-7 pl per oocyte.

### Annexin-V staining in oocytes

Staining was performed with an Annexin-V kit according to the manufacturer's instructions (KenGentec, Nanjing, China). Annexin-V, a phospholipid-binding protein, detects the translocation of phospholipid phosphatidylserine (PS) from the inner to the outer cytoplasmic membrane, which is known to occur during the early stages of apoptosis. At the same time, samples were also stained with propidium iodide (PI) to distinguish live cells from dead cells. Briefly, zona pellucida-free oocytes infected with siRNA adenovirus via co-culturing for 1.5 h and 8 h were washed twice in PBS and stained with 500 μl of binding buffer, which contained 5 μl Annexin-V-fluorescein isothiocyanate (FITC) and 5 μl PI for 5-15 min in the dark. Following this, samples were mounted on siliconized slides and observed under a laser confocal scanning microscope (ZEISS Fluorescent Microsystems, Göttingen, Germany).

### Real time RT-PCR analysis

To determine mRNA abundance, real-time RT-PCR analysis was performed using ABI 7300 (Applied Biosystems, Foster City, CA). RNA isolation was accomplished using the RNeasy Micro Kit (Qiagen, Valencia, CA) from the pooled oocytes (20-25 oocytes/tube). In vitro reverse transcription was carried out using Sensiscript Reverse Transcription Kit (Qiagen) with oligo-dT primer at 37°C for 60 minutes. For the real time RT-PCR reaction, cDNAs were used as templates for amplification to quantify the steady-state mRNA levels of the tested genes using Quanti Tect SYBR Green PCR Kits (Takara Shuzo Co Ltd, Kyoto, Japan). Relative quantitation of target gene expression was evaluated by the 2(-Delta Delta Ct) method [45], and the experiment was repeated at least three times using different sets of oocytes. In our study, beta-actin and GAPDH gene were used as the internal standards referred to similar papers [46,47]. To ensure only target gene sequence-specific, non-genomic products were amplified by real-time RT-PCR, careful design and validation of each primer pair. Primers used for real time RT-PCR are shown in Table 1.

**Table 1:** Primer sequences used for quantitative real-time PCR reactions.

Primer name	Genbank accession number	Primer sequence	Location	Product size (bp)
Hsp27	NM_013560	F: 5'-GCCGCACCAGCCTTCAGC-3'	357-374	147
		R: 5'-CACGCCTTCCTTGGTCTTCACT-3'	482-503	
Caspase 3	NM_009810	F: 5'-ATGGGAGCAAGTCAGTGGAC-3'	128-147	137
		R: 5'-CGTACCAGAGCGAGATGACA-3'	245-264	
Caspase 8	NM_001080126	F: 5'-TGAAGGACAGAAAAGGAACAGA-3'	996-1017	191
		R: 5'-CTTGTCACCGTGGGATAGGATA-3'	1165-1186	
Caspase 9	NM_015733	F: 5'-GGCGGAGCTCATGATGTCTGTG-3'	757-778	269
		R: 5'-TTCCGGTGTGCCATCTCCATCA-3'	1004-1025	
Cytochrome *c*	XR_034017	F: 5'-GGAGGCAAGCATAAGACTGG-3'	219-239	214
		R: 5'-GTCTGCCCTTTCTCCCTTCT-3'	413-432	
Bmp15	NM_009757	F: 5'-CTGACGACCCTACATTGCCCT-3'	467-487	232
		R: 5'-TGTACATGCCAGGAACCTCTGG-3'	677-698	
Gdf9	NM_008110	F: 5'-TCCCAAACCCAGCAGAAGTC-3'	426-445	195
		R:5'-GGAGGAGGAAGAGGCAGAGTTG-3'	609-620	
GAPDH	NM_008084	F: 5'-AGGTTGTCTCCTGCGACTTCA-3'	843-903	216
		R: 5'-GGGTGGTCCAGGGTTTCTTACT-3'	1048-1068	
beta-actin	NM_007393	F:5'-GAGACCTTCAACACCCCAGC-3'	452-471	263
		R:5'-ATGTCACGCACGATTTCCC-3'	696-714	

F, forward; R, reverse

### Immunofluorescence

Oocytes of different treatment groups were fixed in 4% paraformaldehyde in PBS (pH7.4) for at least 30 min at room temperature and then incubated in permeabilization buffer (0.5% Triton X-100 in 20 mM Hepes 3 mM MgCl_2_, 50 mM NaCl, 300 mM sucrose, and 0.02% NaN_3_) for 30 min at 37°C, followed by blocking in 1% BSA for 1 h at room temperature. They were then incubated with Hsp27 antibody (1:100, Santa Cruz, CA), goat polyclonal anti-gdf 9 antibody (1:100, Santa Cruz, CA), rabbit polyclonal anti-cleaved-caspase 3 antibody (1:300; Cell Signaling, Danvers, MA), rabbit polyclonal anti-cleaved-caspase 8 antibody (1:200; Abcam, Cambridge, MA), rabbit monoclonal anti-caspase 9 antibody (1:200; Abcam) and mouse monoclonal cyctochrome *c *antibody (1:100, Santa Cruz) at 4°C overnight. After washing, oocytes were incubated with a fluorescein isothiocyanate (FITC)-conjugated secondary antibody (1:100; Beijing ZhongShan Biotechnology Co., Beijing, China) for 1 h at 37°C, and DNA was counterstained with PI (Sigma). Finally, oocytes were mounted on glass slides with DABCO and examined using ZEISS 510 laser confocal microscopy (ZEISS Fluorescent Microsystems, Göttingen, Germany).

### Western blot analysis

Oocytes of different treatment groups (1000 oocytes/sample) were collected and added immediately into the cell lysis composed of 7 M urea, 2 M thiourea, and 4% CHAPS (W/V) and 1% DTT (W/V), 1% Cocktail (V/V). Proteins were extracted and separated by SDS-PAGE, and transferred onto a polyvinylidene difluoride (PVDF) membrane (GE Healthcare, San Francisco, CA). Following transfer, the membranes were blocked in Tris-buffered saline (TBS) containing 5% skim milk for 1 h at room temperature and then incubated overnight with primary antibodies for cleaved-caspase 3 (1;500; Cell Signalling), cleaved-caspase 8 (1:1000; Abcam), caspase 9 (1:1000, Abcam), Hsp27(1:200; Santa Cruz), and β-tubulin (1:1000; Abcam) at 4°C. After washing 3 times in TBST for 10 min each time, the membrane was incubated for 1 h at 37°C with horseradish peroxidase (HRP)-conjugated secondary antibody (Beijing ZhongShan, Beijing, China). The membrane was then washed 3 times in TBS-Tween (TBST) for 10 min each, and processed using an enhanced chemiluminescence detection system (Alpha Innotech, San Leandro, CA). The molecular weights of the detected proteins were deduced by comparison with recombinant molecular weight standards (New England BioLabs, Ipswich, MA).

### Statistical analysis

Each experiment was repeated at least three times. All data are presented as the mean ± SD, and one-way analysis of variance and a log linear model were used to compare the mRNA and protein levels. Chi-square analysis was used to compare the rates of oocyte maturation and early stage of apoptosis. A value of *P *< 0.05 was considered statistically significant, and if P < 0.01, it was noted.

## Results

### Construction of recombinant adenovirus Ad-shHsp27 and its expression

AD-293 cells were infected by the adenoviruses packaged with hairpin siRNA targeted against Hsp27/control. The results showed that the suppression rate of Hsp27 expression was as high as 75% *vs*. control (Data not shown, *P *< 0.05) after infecting with Ad-shHsp27 and control for 48 h.

**Figure 1:**
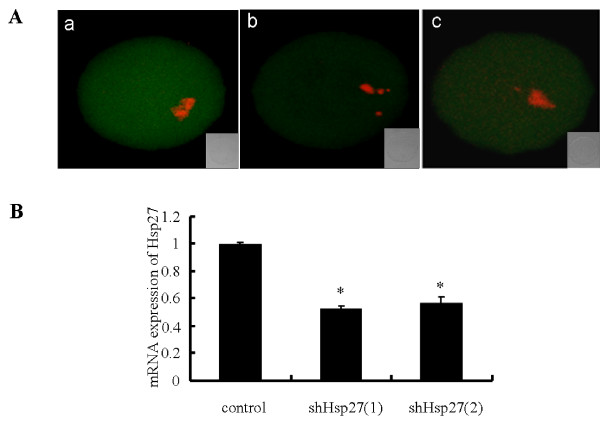
**Ad-shHsp27 effects specific suppression of Hsp27 expression in mouse oocyte**. (A) Immunofluorescence detection of Hsp27 expression after Ad-shHsp27 infection for 48 h. Oocytes were fixed in 4% paraformaldehyde and then stained with anti-Hsp27 antibody (green). Chromosome material was counterstained with propidium iodide (red). a) control-infected oocyte. b) shHsp27(1)-infected oocyte. c shHsp27(2)-infected oocyte. (B) The results of real time RT-PCR showing Ad-shHsp27 infection for 24 h in mouse oocytes. The expression level was calculated from the C_t _values by the 2(-Delta Delta Ct) method, and the mRNA ratio (arbitrary units) of Hsp27 was calculated with respect to that of control. Bar graphs indicate mean ± SD of four replicates. *P < 0.05 *vs*. control.

To examine the reduction degree of Hsp27 mRNA in mouse oocytes after infected with Ad-shHsp27 adenovirus, real time RT-PCR was performed. As Figure 1B displayed, Ad-shHsp27 significantly reduced endogenous Hsp27 mRNA abundance in mouse oocytes (50%). The suppression degree of Hsp27 at protein level was detected by immunofluorescence (Figure 1A), which was in accordance with the real time PCR. These results suggested that the knockdown of Hsp27 expression by Ad-shHsp27 infecting was effective. Therefore, these two siRNA were chosen for the subsequent experiments.

### Localization and expression of Hsp27 in the maturing oocytes

**Figure 2:**
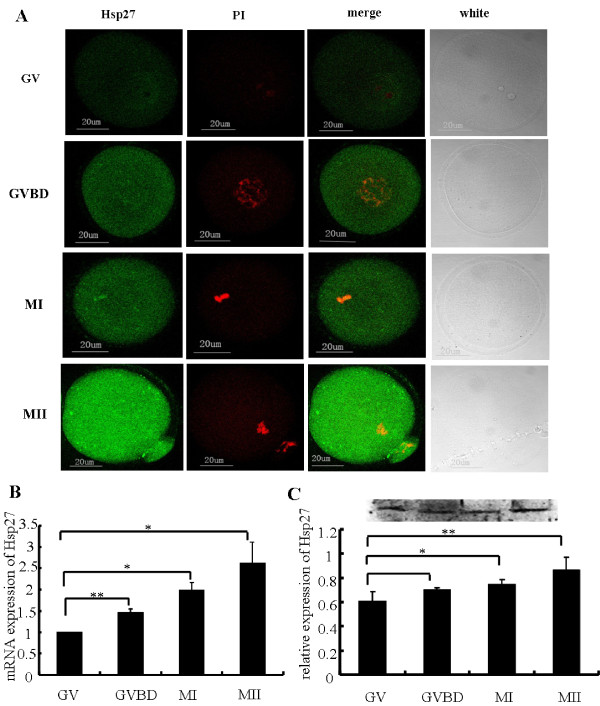
**Localization and expression of Hsp27 in mouse oocytes from GV to MII cultured in vitro**. (A) Immunofluorescence staining of Hsp27 and chromosomes in maturing oocytes. Oocytes culturing for 0 h (GV), 3 h (GVBD), 8 h (MI) and 14 h (MII) *in vitro *were stained with specific Hsp27 antibody (green) and propidium iodide (red). (B) Real time RT-PCR analysis of Hsp27 mRNA in maturing oocytes. Oocytes were cultured *in vitro *for 0, 3, 8 and 14 h. A progressive increase was observed with the maturation of oocytes. (C) Western blot analysis of Hsp27 expression in maturing mouse oocytes. Experiments were repeated at least three times. Bars = 20 μm. *P < 0.05, **P < 0.01 *vs*. control.

The localization of Hsp27 protein in maturing mouse oocytes was showed in Figure 2A. Hsp27 expression was recognized mainly in the cytoplasm and nuclei (except the nucleolus) of mouse oocytes. To assess the levels of Hsp27 mRNA in the maturing oocyte, real time RT-PCR analysis was performed using cDNAs equivalent in oocytes at different developmental stages (Figure. 2B). Hsp27 expression dramatically increased following oocyte maturation, Western blot analysis confirmed that Hsp27 at protein level also increased following oocyte maturation, and dramatically increased in MII (Figure. 2C), which was consistent with the results of real time RT-PCR and immunofluorescence.

### Microinjection of Hsp27 antibody into mouse oocytes

Polyclonal antibody of Hsp27 was microinjected into oocytes cytoplasm at GV stage to downregulate the activity of Hsp27. After 3 h of microinjection, the GVBD rate (77.0%) significantly increased, when compared with normal control (67.3%) or IgG-injected control (64.4%) (*P *< 0.01, *vs *IgG-injected group). After 14 h of microinjection, the maturation rate of MII stage (67.3%) was also higher than those of two controls (below 60%) (*P *< 0.01, *vs *IgG-injected group; Table 2). These findings suggested that the lowed Hsp27 activity in mouse oocytes could improve the maturation of oocytes.

**Table 2:** Maturation of mouse oocytes after microinjecting of Hsp27 antibody at 3 h and 14 h of *in vitro *culture.

	No. of oocytes (%)		
Treatment	Total	Germinal vesicle breakdown	Metaphase II
Control	312	210(67.3)	186(59.6)
IgG	323	208(64.4)	178(55.1)
Antibody	365	281(77.0)**	246(67.3)**

** Values are statistical significance at *P *< 0.01(*vs*. IgG).

### Infection of Ad-shHsp27 adenovirus into pellucida-free GV oocytes

Oocyte maturation of mouse zona-free GV oocytes was significantly improved by infecting with Ad-shHsp27, when compared with negative control at GVBD stage and MII stage (Table 3). The maturation rates of GVBD stage in shHsp27 (1), shHsp27 (2) groups were 86.0% and 88.7%, which were higher than control (76.2%) (*P *< 0.01). Accordingly, compared with control (60.8%), the ratios of MII stage in shHsp27 (1) and shHsp27 (2) groups (76.4% and 73.8%) were also increased dramatically(*P *< 0.01). Same outcome was found in the antibody microinjection (Table 2).

**Table 3:** Stages of oocyte nuclear maturation from the different treatment groups after 3 h and 14 h of IVM.

	No. of oocytes (%)		
Treatment	Total	Germinal vesicle breakdown	Metaphase II
control	319	243(76.2)	194(60.8)
shHsp27(1)	336	298(88.7)**	257(76.4)**
shHsp27(2)	314	270(86.0)**	232(73.8)**

** Values are statistical significance at *P *< 0.01(*vs*. control).

### Annexin-V staining of oocytes

**Figure 3:**
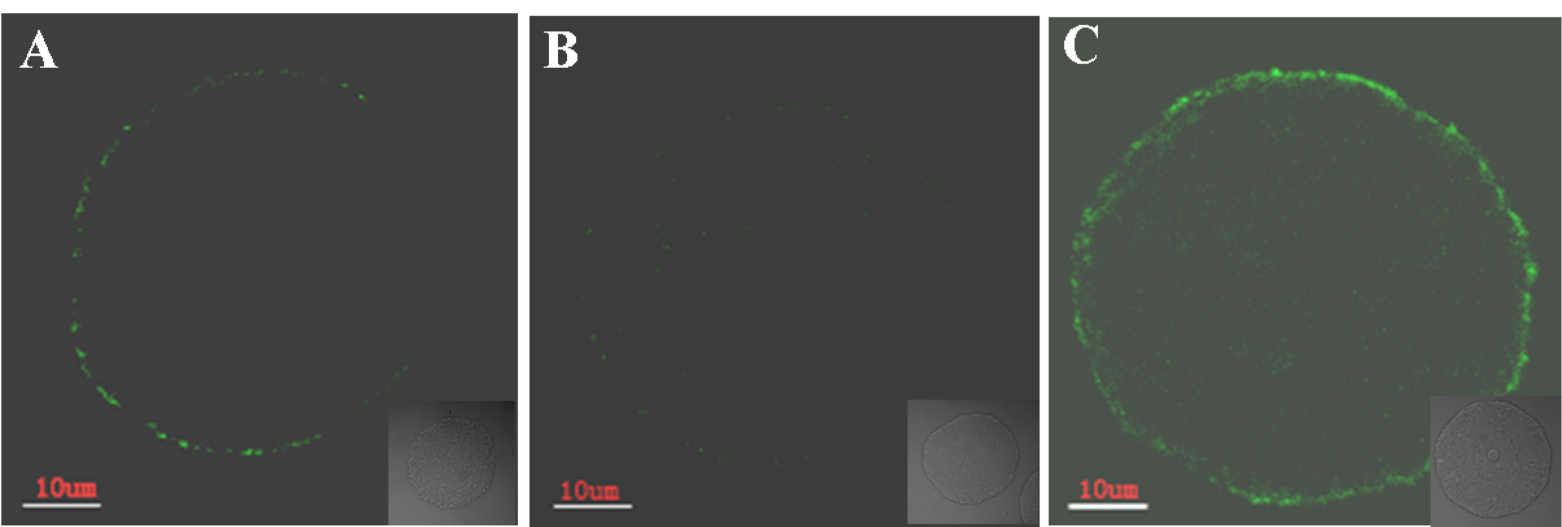
**Oocyte classification by Annexin-V staining**. (A) necrotic oocytes. Discontinuous green signal was originated from the remnant portions of the membrane. (B) Oocyte Annexin-V negative: no signal in the cytoplasmic membrane. (C) Annexin-V positive (early stage of apoptosis): a clear green signal is observed in the oocyte membrane. Bars = 10 μm.

Oocytes were classified into three groups as Anguita *et al *described [48]. The first group contained necrotic oocytes with PI-positive red nuclei, indicative of membrane damage. Oocytes with a discontinuous green signal originating from the remnant portions of the membrane were viable non-apoptotic oocytes (Figure. 3A). The second group contained viable oocytes which were negative for Annexin-V staining (Figure. 3B). The third group consisted of early apoptotic oocytes with homogeneous Annexin-V-positive signal in the membrane (Figure. 3C).

The ratios of Annexin-V-positive cells in two shHsp27 groups were higher than negative control (Table 4). After 3 h of treatment, the early apoptotic rate of oocytes infected with Ad-shHsp27 (38.9%, 36.2%) was not significantly different from control (33.8%). The ratios of early apoptotic oocytes in shHsp27-treated groups after 8 h of treatment were 46.5% and 45.6%, which were significantly higher than the negative control (34.1%). The results suggested that Hsp27 downregulation in oocytes could promote the early stage of apoptosis.

**Table 4:** The rate of early stage of apoptosis in oocytes at 3 and 8 hours after siRNA adneovirus infection, was evaluated by Annexin-V staining in different groups (Control, shHsp27(1), shHsp27(2)).

	3 h		8 h	
Treatment	Total	No. of early apoptosis oocytes (%)	Total	No. of early apoptosis oocytes (%)
control	130	44 (33.8)	129	44 (34.1)
shHsp27(1)	131	51 (38.9)	131	61 (46.5)*
shHsp27(2)	149	54 (36.2)	125	57 (45.6)*

3 h and 8 h indicated the time of culture *in vitro *after adenovirus infection.

*Significant changes within group (* *P *< 0.05 *vs*. control).

### Variation of downstream apoptosis-related factors and oocyte secreted factors with defect of Hsp27 protein

**Figure 4:**
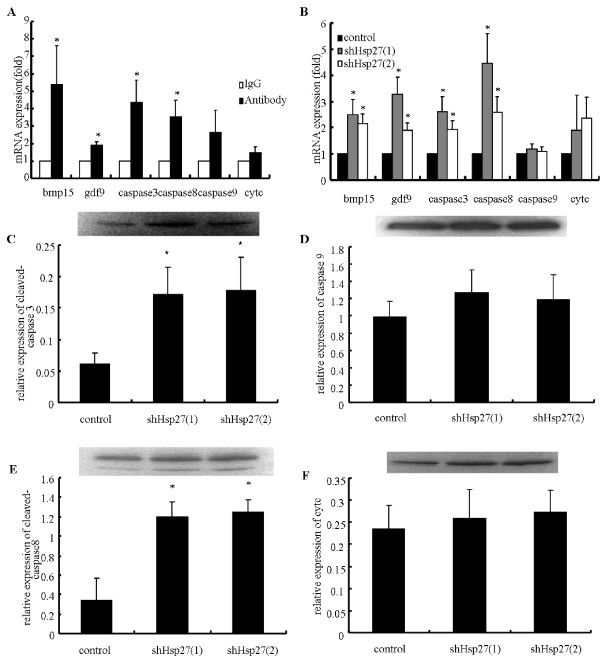
**Effect of Hsp27 downregulation in GV oocytes on the expression of some key apoptotic proteins and oocyte-derived growth factors**. (A) Real time RT-PCR analysis in mouse oocytes after microinjecting Hsp27 antibody. Oocytes were cultured for 18-24 h. (B) Real time RT-PCR analysis in oocytes after infection with Ad-shHsp27. (C-F) Western blot analysis of key apoptotic factors in oocytes after infection with Ad-shHsp27. Measurements were plotted as the mean of at least three biological replicates ± SD. Changes are labeled as significant (*) if *P *< 0.05.

**Figure 5:**
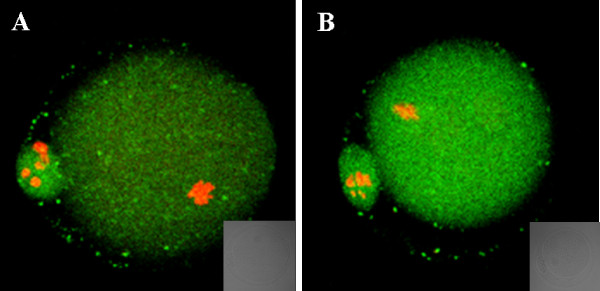
**Immunofluorescence staining of cleaved-caspase 3 in oocytes after microinjecting of Hsp27 antibody (The other factors not shown)**. Oocytes were fixed in 4% paraformaldehyde after microinjecting of Hsp27 antibody for 36-48 h. Then these oocytes were stained with cleaved-caspase 3 antibody (green). Chromosome material was counterstained with propidium iodide (red). A) IgG-injected oocyte. B) Hsp27 antibody-injected oocyte.

To elucidate the involved apoptotic pathway(s), the expressions of four apoptotic factors (caspases 8, caspase 9, caspase 3 and cytochrome *c*) were measured in oocytes following different treatment by real time RT-PCR, immunofluorescence and western blot. As shown in Figure 4 and Figure 5, the results of these assays were accordant. Hsp27 downregulation in mouse oocytes dramatically increased the activitied caspase 8 and caspase 3, while caspase 9 and cytochrome *c *activities did not change. These results suggested that downregulation of Hsp27 in mouse oocytes resulted in activation exclusively of the extrinsic, caspase 8-mediated apoptotic pathway, rather than the intrinsic, caspase 9-mediated pathway.

In addition, the expressions of two important oocyte-derived growth factors (bmp 15 and gdf 9) after downregulation of Hsp27 in mouse oocytes were also investigated (Figure. 4A and 4B). The results indicated that bmp 15 and gdf 9 were increased significantly in oocyte after downregulation of Hsp27.

## Discussion

In the present study, the effect of Hsp27 downregulation on oocyte development and maturation was investigated in the mouse oocyte model cultured *in vitro*. Expression of Hsp27 gene was downregulated in oocytes using siRNA adenovirus infection, while the activity of Hsp27 was decreased by microinjection of polyclonal Hsp27 antibody into mouse GV oocytes. Interestingly, our results showed that Hsp27 downregulation in mouse oocytes improved oocyte maturation and increased oocyte early stage of apoptosis.

Hsp27 was investigated as an antiapoptotic factor [49-51]. Hsp27 could directly bind and co-precipitate with cytochrome *c*, consequently leading to activation of the caspase 9 and caspase 3 in mouse, rat and human cells [31,33,52,53]. Kamradt *et al *reported that Hsp27 could negatively regulate cytochrome *c*- and caspase 8-dependent activation of caspase 3 in human breast carcinoma cells [54]. To our knowledge, there were few reports about the roles of Hsp27 in oocyte. In this pilot study, after downregulation of Hsp27 in mouse oocytes, the ratio of early stage of apoptosis was significantly increased. Meanwhile, the expression of major apoptotic factors i.e. caspase 3, caspase 8 was dramatically increased. These results suggested that downregulation of Hsp27 in mouse oocytes might increase early stage of apoptosis by inducing the activation of the extrinsic, caspase 8-mediated apoptotic pathway.

Our results showed that the lowed levels of Hsp27 significantly increased GVBD rate and MII rates in both siRNA infection group and antibody microinjection group when compared with controls, indicating that Hsp27 downregulation was positively related to oocyte maturation. This result was also supported by detecting the expression of two important oocyte-derived growth factors-bmp 15 and gdf 9, which were known to be responsible for controlling fundamental physiological processes in oocyte development and follicular growth [55]. Juengel *et al *reported that immunization against gdf 9 and bmp 15 alone or together in cattle oocytes could block folliculogenesis and reduce follicular size, which indicated the critical role of bmp 15 and gdf 9 on oocyte development and follicular growth [56]. In this study, we found that bmp15 and gdf 9 were noticeably upregulated after Hsp27 downregulation in the mouse oocytes model cultured *in vitro*, suggesting that there was an enhanced effect of downregulated Hsp27 on oocyte development.

Recently, there were numerous studies focused on the relation between early stage of apoptosis and oocyte developmental competency in the pooled oocytes cultured *in vitro *[12,48,57,58]. As we known, early stage of apoptosis was regarded as a sequential, but reversible, process of cell death [10,57]. As Anguita *et al *and Jaroudi *et al *indicated, the oocytes undergoing early stage of apoptosis did not mean that they must develop into late apoptosis, early stage of apoptosis oppositely decreased their developmental competence [48,59]. Bilodeau-Goeseels and Panich proved that oocytes with early signs of atresia had good developmental potential by detecting the transcriptional activity in early bovine embryos from different classes of cumulus-oocyte complexes [60]. Additionally, some clinical studies provided consistent results that oocytes from slightly atretic COCs with signs of cumulus expansion had a better embryonic developmental capacity after IVF than those considered to be of the highest quality [61-65]. Concordantly, the percentage of apoptotic cumulus cells increased when COCs were exposed to FSH. Oocytes from those COCs exhibited a higher developmental potential in terms of blastocyst formation rate [66-68]. Those reports suggested that early stage of apoptosis had a positive relation with the improved oocyte developmental potential. In our present study, downregulation of Hsp27 led to a lower antiapoptotic effect and a significant increase in the GVBD rate and MII rate, which was consistent with previous reports [57]. Taking these results together, we speculated that downregulation of Hsp27 induced early oocyte apoptosis, consequently improving oocyte development. In fact, it is difficult at present to understand the molecular mechanism of the correlation between early stage of apoptosis and subsequent maturation of oocyte in follicles in PCOS. Our hypothesis is that downregulation of Hsp27 could be one of the bridges between those events, which is our objectives in future studies.

Previous proteomic analysis comparing normal and PCOS ovarian protein profiles identified that expression of Hsp27 in PCOS ovaries was decreased when compared with normal ovarian tissues [35]. PCOS is known as the most common cause of anovulatory infertility, resulting from a disorder of follicular maturation of uncertain aetiology [15]. Several reports showed that apoptosis was perturbed in PCOS ovaries, with overexpression of apoptotic factors and downregulation of anti-apoptosis factors. However, the others showed the reversed results [69-71]. Das *et al *reported that there were significant differences in cell death rate and proliferation rate of granulosa cell populations in PCOS patients [72,73]. Oocyte development is interdependent with its surrounding granulose cells[11], and disfunction of granulose cells may contribute to the abnormal folliculogenesis observed in PCOS [5,74,75]. Interestingly, we found in this study that the downregulated Hsp27 improved oocyte maturation, which seems to be contradicted with the atresia follicles observed in PCOS. One possible explanation for this contradiction could be that our research was performed in mouse oocyte model cultured *in vitro*. This model simulates the normal oocyte's development, which could be different from the pathological procedure of PCOS characterized by an abundance of large but immature follicles. We need more studies on the effects of Hsp27 in PCOS pathophysiology in future studies.

## Conclusions

In conclusion, evidence provided in the present study indicated that downregulation of Hsp27 improved oocyte maturation, and increased early apoptosis of mouse oocytes by triggering the extrinsic, capsase 8-mediated pathway. This is the first report, to our knowledge, to establish a direct link between Hsp27 and oocyte maturation. Relative deficiency of Hsp27 expression in GV oocytes may contribute to the disordered oocyte development in PCOS. Further studies will be required to elucidate the role of Hsp27 in oocyte maturation in vivo and PCOS pathophysiology.

## Competing interests

The authors declare that they have no competing interests.

## Authors' contributions

JJL carried out the main experiments and wrote the first draft of the manuscript. LBC performed the antibody microinjection. XM, YGC and JYL designed the study and proofread the final manuscript. All authors read and approved the final manuscript.
